# Acute to Subacute Atraumatic Entrapment Neuropathies in Patients With CMT1A: A Report of a Distinct Phenotypic Variant of CMT1A

**DOI:** 10.3389/fneur.2022.826634

**Published:** 2022-02-25

**Authors:** Zhiyong Chen, Monica Saini, Shermyn X. M. Neo, Peng-Soon Ng, Jasmine S. Koh, Kalpana Prasad, Kamal Verma, Sonia Davila, Weng Khong Lim, Ziqun Phua, Michelle M. Li, Corrine Kang, Karine S. S. Tay, Josiah Y. H. Chai

**Affiliations:** ^1^Department of Neurology, National Neuroscience Institute, Singapore, Singapore; ^2^Singhealth Duke-National University of Singapore (NUS) Institute of Precision Medicine, Singapore, Singapore; ^3^Cardiovascular and Metabolic Disorders, Duke-National University of Singapore (NUS) Medical School, Singapore, Singapore; ^4^SingHealth Duke-National University of Singapore (NUS) Genomic Medicine Centre, Singapore, Singapore; ^5^Cancer and Stem Cell Biology Program, Duke-National University of Singapore (NUS) Medical School, Singapore, Singapore; ^6^Neurodiagnostic Laboratory, National Neuroscience Institute, Singapore, Singapore; ^7^Clinical Measurement Unit, Changi General Hospital, Singapore, Singapore; ^8^Neuromuscular Laboratory, National Neuroscience Institute, Singapore, Singapore

**Keywords:** Charcot-Marie-Tooth type 1A (CMT1A), CMT1A duplication, PMP22 protein, peripheral nerve ultrasound, Charcot-Marie-Tooth disease

## Abstract

Charcot-Marie-Tooth type 1A (CMT1A) is typically characterised as a childhood-onset, symmetrical, length-dependent polyneuropathy with a gradual progressive clinical course. Acute to subacute neurological deterioration in CMT1A is rare, and has been reported secondary to overlap pathologies including inflammatory neuropathy. We identified two patients with CMT1A who presented with acute to subacute, atraumatic, entrapment neuropathies as an initial symptom. A superimposed inflammatory neuropathy was excluded. Both patients had a diffuse demyelinating polyneuropathy, with markedly low motor nerve conduction velocities (<20 m/s). In both patients, we demonstrated symptomatic and asymptomatic partial conduction blocks at multiple entrapment sites. Nerve ultrasound findings in our patients demonstrated marked diffuse nerve enlargement, more pronounced at non-entrapment sites compared to entrapment sites. We discuss ways to distinguish this condition from its other differentials. We propose pathophysiological mechanisms underlying this condition. We propose that CMT1A with acute to subacute, atraumatic, entrapment neuropathies to be a distinct phenotypic variant of CMT1A.

## Introduction

CMT1A is an autosomal dominant, demyelinating polyneuropathy caused by a 1.4 Mb duplication on chromosome 17p11.2. It is the commonest form of Charcot-Marie-Tooth (CMT) disease. While the disease spectrum is broad, it is typically characterised by a childhood-onset, symmetric, length-dependent polyneuropathy associated with distal wasting, weakness, and sensory loss that is gradually progressive over decades (1).

Up to 7% of patients with CMT1A may have slightly asymmetric neurological deficits (2). Acute to subacute neurological deterioration is rare (3), and has been reported in superimposed inflammatory demyelinating neuropathy, radiculopathy secondary to degenerative spinal disease (2, 4–6), or following exposure to neurotoxic agents (7).

Here, we report two patients with CMT1A who presented with acute to subacute asymmetric neuropathy with electrophysiological features suggestive of entrapment neuropathy [focal demyelination]. No congruent history of compression or minor trauma was evident, and a superimposed inflammatory neuropathy was excluded. As such clinical presentations may be confused with chronic inflammatory demyelinating polyneuropathy [CIDP], or overlap hereditary and inflammatory demyelinating neuropathy, we evaluate the role of ancillary investigations, such as nerve ultrasound, in the diagnosis of this clinical entity, suggest strategies to avoid misdiagnosis, with the aim of avoiding unnecessary immunosuppressive therapy for patients with this unique clinical syndrome. We also propose potential pathophysiological mechanisms underlying this condition.

## Methods

The neuromuscular database is a prospective registry of patients with various neuromuscular disorders, diagnosed by neuromuscular specialists, at the National Neuroscience Institute and Changi General Hospital. Following the identification of the index case [Patient 1], the neuromuscular database was probed for identification of patients fulfilling the following criteria:

1. Genetically proven duplication of the *PMP22* gene.
2. Acute to subacute, stepwise, progressive weakness and/or sensory dysfunction of the extremities.
3. Exclusion of chronic inflammatory demyelinating polyradiculoneuropathy (CIDP), as per European Federation of Neurological Societies and Peripheral Nerve Society CIDP clinical diagnostic criteria (8).
4. Electrophysiological evidence of focal demyelination or conduction block (9).

Clinical data of identified patients was reviewed. Collated data included clinical characteristics as well as electrophysiological tests, magnetic resonance imaging (MRI), peripheral nerve ultrasound and treatment received.

Electrophysiological studies were performed using Nicolet Viking and Nihon Kohden electromyography (EMG) devices. Partial conduction block was defined based on consensus guidelines (9). The measurement of area of compound muscle action potential (CMAP) is performed by measuring the area under the curve of the CMAP waveform from the first negative deflection to its first baseline crossing from negative to positive, relative to the baseline. The presence of a partial conduction block is only assessed for if the negative-peak amplitude of the CMAP with distal stimulation is 20% or more of the lower limit of normal. Details regarding the cut-off diagnosis of probable and definite conduction block is detailed in Supplementary Table 2. A EPIQ 7G (Philips Healthcare, Amsterdam, The Netherlands) ultrasound system with 5–18 MHz transducers were used for nerve high resolution ultrasound (HRUS). Nerve enlargement was assessed qualitatively by longitudinal assessment along the length of the nerve, and quantitatively by measuring the cross section area (CSA) (10). Vascularity was assessed qualitatively by colour doppler. CSA was measured at pre-determined sites: median nerve (palm, wrist, and forearm), ulnar nerve (wrist, below elbow, elbow, above elbow), radial nerve (spiral groove), sciatic nerve (bifurcation), tibial nerve (bifurcation, proximal calf, ankle), peroneal nerve (bifurcation, fibular head), sural nerve (ankle) (10). CSA was calculated by continuous manual tracing of the nerve circumference excluding the hyperechoic epineurial rim (11). Cut-off values for abnormal CSA were derived from normative data from our laboratory and defined as values above the upper limit (i.e., mean + 2 standard deviation [SD]) (12).

This study was approved by the Singapore Health Services Centralised Institutional Review Board (CIRB 2018-2341). Written informed consent was obtained from all patients.

## Molecular Analysis

Whole-exome sequencing (Agilent SureSelect V6) was performed on extracted genomic DNA to a target sequencing depth of 100X. The resulting sequencing reads were aligned to the human reference genome (GRCh37) using BWA (13), and then processed using GATK 4.2.0 (14) according to prescribed best practises. Variants that were identified in the process were then annotated with information such as genomic region, affected gene(s), variant effect on coding sequence, population allele frequencies and *in silico* pathogenicity scores using ANNOVAR (15). At the same time, putative copy number variants (CNVs) were identified using panelcn.MOPS (16). The presence of a *PMP22* duplication was determined by comparing exon-level and gene-level coverage against a panel of control samples, and identifying samples that have abnormally high coverage in the gene (Supplementary Figure 1). Sixty genes known to cause CMT were additionally analysed for the presence of genetic variants (Supplementary Table 3). The pathogenicity of an identified genetic variant was evaluated using the Varsome bioinformatic tool (https://varsome.com/).

## Case Description

We identified two patients with genetically proven CMT1A, with acute to subacute neurological presentation. Patient case summaries are as below.

### Patient 1

Patient 1 was a Chinese female who presented, at 28 years of age, with an acute onset, painful left lower limb weakness [time to nadir of symptoms: 2 weeks], 3 months prior to the first medical consult. This was associated with intermittent numbness and weakness of bilateral hands. There was no preceding injury or abnormal postures. Developmental milestones were normal. The patient reported difficulty in dorsiflexing her feet, most noticeable when swimming in her teens. She was unable to pass physical fitness tests since secondary and high school. She also reported occasional ankle sprains since 23 years of age. She has a younger brother aged 25, who was well. There was no parental consanguinity. The patient's mother reported prior ankle sprains, without obvious foot abnormalities.

**Figure 1:**
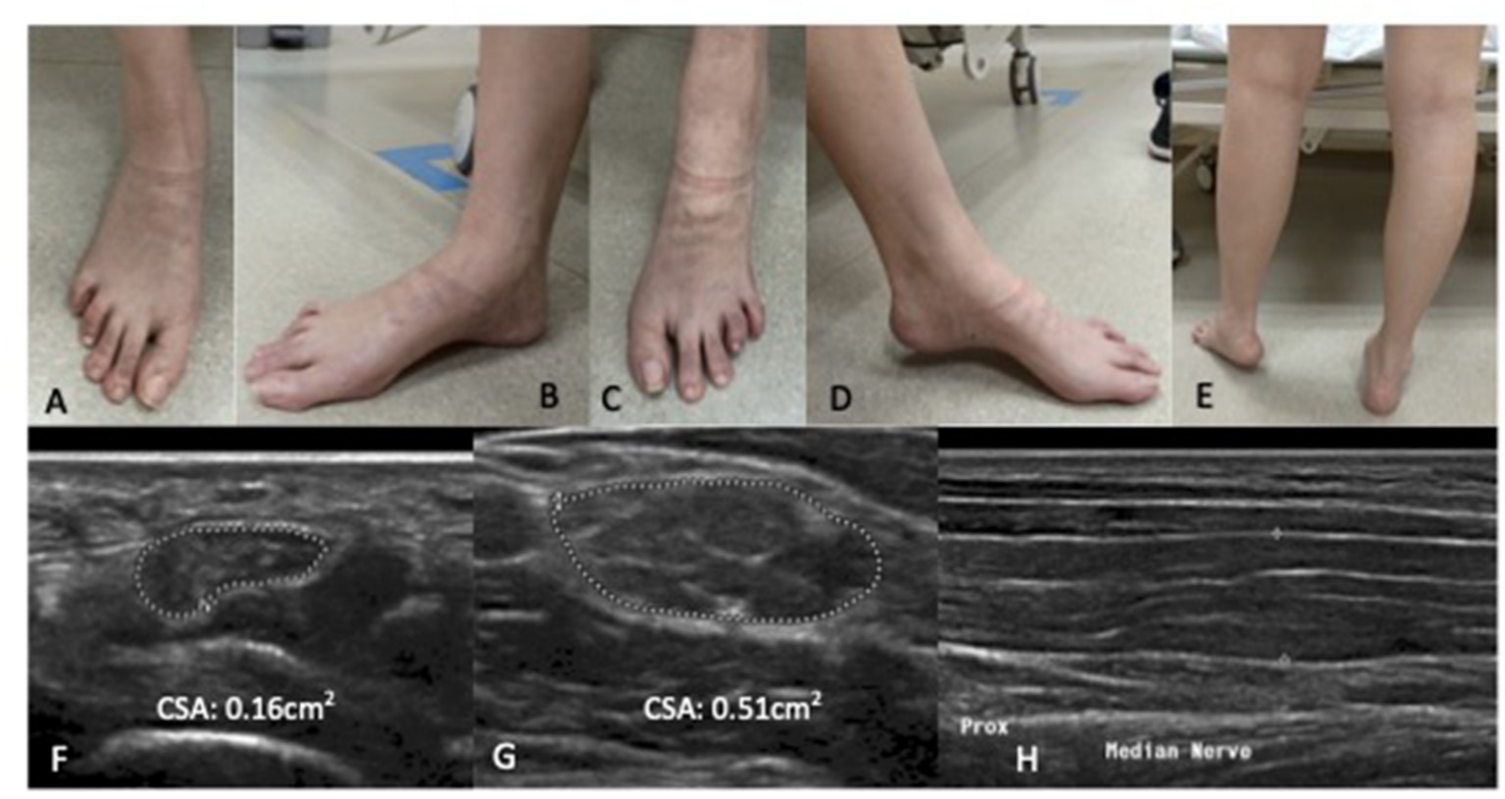
Patient one: Bilateral wasting of intrinsic foot muscles, mild elevation of bilateral plantar arches with claw toes. Left foot drop secondary to left common peroneal neuropathy at the fibular head. There is no significant calf wasting **(E)**. **(A,B)** right foot; **(C,D)** left foot. High resolution ultrasound of the right median nerve findings at the carpal tunnel **(F)**, at the elbow **(G)** showing marked diffuse increase cross sectional area more than 3× upper amit normal at non- entrapment sites with less marked enlargement at entrapment sites **(H)**. **(F,G)** Transverse section; **(H)** Longitudinal section.

Examination revealed symmetrical mild wasting of intrinsic muscles of the feet, mild elevation of bilateral plantar arch and toe clawing (Figure 1). Upper limb power was normal, apart from mild weakness [MRC grade 4/5] of left finger and thumb abduction. There was severe left foot drop [left ankle dorsiflexion MRC 1/5], while bilateral ankle plantar flexion and bilateral hip abduction was full. Deep tendon reflexes were diffusely absent. Sensation to all modalities was reduced in a glove and stocking pattern. Bilateral superficial radial nerves at the anatomical snuffbox, superficial peroneal nerves, common peroneal nerves at the fibular head, ulnar nerves at the elbow were distinctly palpable.

Nerve conduction study (Table 1A) revealed a generalised, severe reduction in nerve conduction velocities (NCV < 15 m/s) in both upper and lower limbs. Superimposed multiple conduction blocks, without temporal dispersion, were noted over entrapment sites. Sensory responses were diffusely absent. Cerebrospinal fluid (CSF) analysis was normal. Peripheral nerve ultrasound revealed diffuse and marked nerve enlargement [> x3 upper limit of normal (ULN)] across non-entrapment sites; this was less pronounced across entrapment sites (Figure 1 and Table 2). Colour doppler did not show increase in nerve vascularity.

**Table 1:** Electrophysiological findings for patient one (A) and patient two (B).

			**Right**							**Left**				
	**DL (ms)**	**CMAP (mV)**	**Normal CMAP range (mV)**	**Area (mVms)**	**Duration (ms)**	**MNCV (m/s)**	**Type of partial conduction block/% area loss**	**DL (ms)**	**CMAP (mV)**	**Normal CMAP range (mV)**	**Area (mVms)**	**Duration (ms)**	**MNCV (m/s)**	**Type of partial conduction block/% area loss**
**(A)**														
**Median (APB)**														
Wrist	12.0	3.4	>8.0	18.1	9.6			13.4	4.2	>8.0	22.9	10.4		
Elbow		2.7		12.6	8.9	15			3.3		15.9	11.2	15	
**Ulnar (ADM)**														
Wrist	8.7	6.9	>6.0	34.5	9.7			9.3	4.2	>6.0	24.1	10.3		
*Below elbow*		**6.2**		**31.0**	10.1	13			**3.6**		**20.8**	10.8	15	
*Above elbow*		**3.9**		**18.9**	10.4	14	**Probable/39**		**1.0**		**6.4**	13.7	14	**Definite/69**
Axilla		3.5		17.9	10.1				0.9		5.3	14.0		
**Ulnar (FDI)**														
Wrist	14	4.3		19.2	9.3			11.5	4.4		16.9	8.4		
*Below elbow*		**3.8**		**17.5**	10.5	14			**3.7**		**15.3**	8.8	15	
*Above elbow*		**2.0**		**10.5**	10.7	14	**Definite/40**		**0.6**		**2.6**	11.0	13	**Definite/83**
Axilla		1.5		7.4	11.8				0.4		1.3	10.7		
**Radial (EIP)**														
Forearm	4.4	1.9	>3.5	13.5	14.8			4.2	1.6	>3.5	10.8	15.1		
Lateral brachium		1.5		10.3	17.8	15			1.2		8.3	18.3	16	
*Below spiral groove*		**1.3**		**9.0**	18.3	15			**0.9**		**6.8**	20.0	15	
*Above spiral groove*		**0.1**		**1.1**	20.7	11	**Probable/88**		**0.3**		**2.2**	18.3	12	**Probable/68**
**Tibial (AH)**														
Ankle	19.0	0.1	>7.0	1.0	20.3			Ab-sent		>7.0				
**Peroneal (EDB)**														
Ankle	12.3	1.5	>4.0	7.7	9.1			11.6	0.7	>4.0	2.3	11.4		
*Fibula (head)*		**1.0**		**5.7**	11.7	14								
*Above knee*		**0.4**		**2.7**	13.9	13	**Definite/53**							
**Peroneal (TA)**														
*Fibula (head)*	8.4	**1.9**	>2.1	**16.7**	16.4			9.1	**1.6**	>2.1	**14.1**	15.4		
*Above knee*		**0.6**		**5.1**	19.1	16	**Definite/70**		**0.2**		**0.2**	14.0	9	**Definite/98**
**(B)**														
**Median (APB)**														
wrist	15.9	0.6	>8.0	2.1	7.0			8.9	2.9	>8.0	15.6	11.7		
elbow		0.4		1.3	7.9	18			2.8		12.7	12.7	16.7	
**Ulnar (ADM)**														
wrist	7.8	1.7	>6.0	7.7	8.2			8.8	1.1	>6.0	5.0	8.2		
Below elbow		1.5		6.5	9.1	17			0.9		4.2	10.9	20	
Above elbow		1.1		5.5	8.8	17			0.7		3.9	12.0	18	
**Radial (EIP)**														
Forearm	4.5	1.5	>3.5	7.9	11.5			8.5	1.8	>3.5	10.2	12.8		
*Lateral brachium*		**1.6**		**8.8**	13.2	15			**1.0**		**5.2**	13.8		**Probable/49**
*Above spiral groove*		**0.8**		**4.4**	13.5	18	**Probable/50**		**0.6**		**4.4**	16.7		
**Tibial (AH)**														
Ankle	Ab-sent		>6.0					Ab-sent		>6.0				
**Peroneal (TA)**														
*Fibula (head)*	7.8	**1.1**	**>1.5**	**11.8**	15.2			7.2	**0.8**	**>1.5**	**7.5**	18.8		
*Above knee*		**0.7**		**4.3**	16.0	10	**Definite/64**		**0.2**		**1.5**	20.0	11	**Definite/80**

*There is marked slowing of motor nerve conduction velocity of <20 m/s with definite to probable partial conduction blocks across multiple entrapment sites for patient one and entrapment as well as non-entrapment sites for patient two. Nerve segments whereby partial motor conduction blocks are observed are in bold. Normative distal CMAP values are age and height-matched*.

*ADM, abductor digitorum minimi; AH, abductor hallucis; APB, abductor pollicis brevis; CMAP, compound motor action potential; Definite, definite partial conduction block; DL, distal latency; EIP, extensor indicis propius; FDI, first dorsal interosseus; MNCV, motor nerve conduction velocity; Probable, probable partial conduction block; TA, tibialis anterior*.

**Table 2:** Nerve ultrasound findings for patients one and two.

**Nerve Site**	**Patient one**		**Patient two**		
	**Cross section area (cm** ^**2**^ **)**		**Cross section area (cm** ^**2**^ **)**		
**Upper extremity**	**Left**	**Right**	**Left**	**Right**	**Reference range (cm^**2**^)**
**Median**					
Carpal tunnel exit	NA	0.19	0.14	0.13	<0.12
Carpal tunnel at wrist crease	NA	0.16	0.17	0.15	<0.12
5 cm proximal to wrist crease	NA	0.42	0.31	0.32	
Forearm	NA	**0.50**	**0.30**	**0.32**	<0.08
**Ulnar**					
Wrist crease	NA	0.15	NA	0.07	<0.06
Forearm	NA	NA	NA	0.22	
Distal elbow	NA	**0.31**	NA	0.16	<0.07
Elbow	NA	0.16	NA	0.13	<0.10
5 cm proximal to elbow	NA	**0.50**	NA	**0.26**	<0.07
**Radial**					
Superficial radial at wrist	NA	0.08	NA	NA	
Elbow	NA	0.58	NA	NA	
**Lower extremity**					
**Sciatic**-Thigh	1.30	NA	NA	1.31	<0.47
**Tibial**-at Popliteal Fossa	**1.16**	NA	NA	**0.71**	<0.24
**Peroneal**-at sciatic bifurcation	**0.58**	NA	NA	**0.51**	<0.13
**Peroneal-**at Fibular head	**0.31**	NA	0.26	0.14	<0.09
**Sural**-at Ankle	**0.22**	NA	NA	0.08	<0.04

*Areas where there is enlargement of nerve cross section area more than three times upper limit of normal are in bold*.

In view of an acute-subacute presentation, superimposed on a chronic clinical course, with electrophysiological evidence of non-uniform demyelination, the diagnosis of overlap inherited neuropathy and chronic inflammatory demyelinating polyneuropathy [CIDP] was considered. Patient received one cycle of intravenous immunoglobulin [2 g/kg], without significant clinical benefit before genetic test returned. Genetic testing revealed duplication of the *PMP22* gene. No other pathogenic variants in genes known to cause Charcot-Marie-Tooth disease were noted. Examination of patient's mother revealed a symmetric, length-dependent polyneuropathy associated with mild distal lower limb wasting. Electrophysiological and genetic studies were not performed on patients mother.

Patient was prescribed with left foot orthosis and initiated on physical therapy. These interventions resulted in reduced functional disability. There was no improvement in asymmetric foot drop 6 months from onset of acute neurological symptoms. The final diagnosis was CMT1A with acute atraumatic left common peroneal neuropathy at the fibular neck.

### Patient 2

Patient 2 was a 46-year-old Chinese man who presented after a fall. He had transient numbness and cramps of bilateral hands 12 months prior to the first medical consult. This was followed by painless left lower limb weakness, followed 1 month later by right lower limb weakness. Bilateral lower limb weakness reached a nadir over 2 months, resulting in an inability to run. He had previously been physically active, engaging regularly in recreational sports running up to five kilometres. Perinatal history and developmental milestones were unremarkable, and there was no parental consanguinity. He is the second of four siblings, who were all well. One niece [brother's daughter] was reported to have developed difficulties with ambulation at the age of 3 years.

Examination revealed mild symmetrical wasting of intrinsic muscles of the feet, with mild elevation of bilateral plantar arch. Power in the upper limbs was full [MRC grade 5/5] except for weakness of bilateral finger abduction [MRC grade 3/5] and thumb abduction [MRC grade 4/5]. There was bilateral foot drop, [right ankle dorsiflexion MRC grade 3/5, left ankle dorsiflexion MRC grade 0/5], as well as bilateral foot eversion [right MRC grade 3/5, left MRC grade 0/5] and toe extension weakness [right MRC grade 3/5, left MRC grade 0/5]. Deep tendon reflexes were 1+ in both upper and lower limbs. Vibration and proprioceptive sense in bilateral big toes was absent. Pinprick sensation was reduced up to bilateral ankles. Bilateral superficial radial nerves at the anatomical snuffbox, superficial peroneal nerves, common peroneal nerves at the fibular head, ulnar nerves at the elbow were enlarged.

Nerve conduction study showed revealed generalised and significant reduction in nerve conduction velocities in upper and lower limbs (NCV < 20 m/s). Superimposed multiple conduction blocks, without temporal dispersion, were noted over multiple entrapment and non-entrapment sites in the left radial nerve (Table 1B). Sensory responses were diffusely absent. In addition, needle electromyography suggested a left common peroneal neuropathy at the fibular head (Supplementary Table 1).

**Figure 2:**
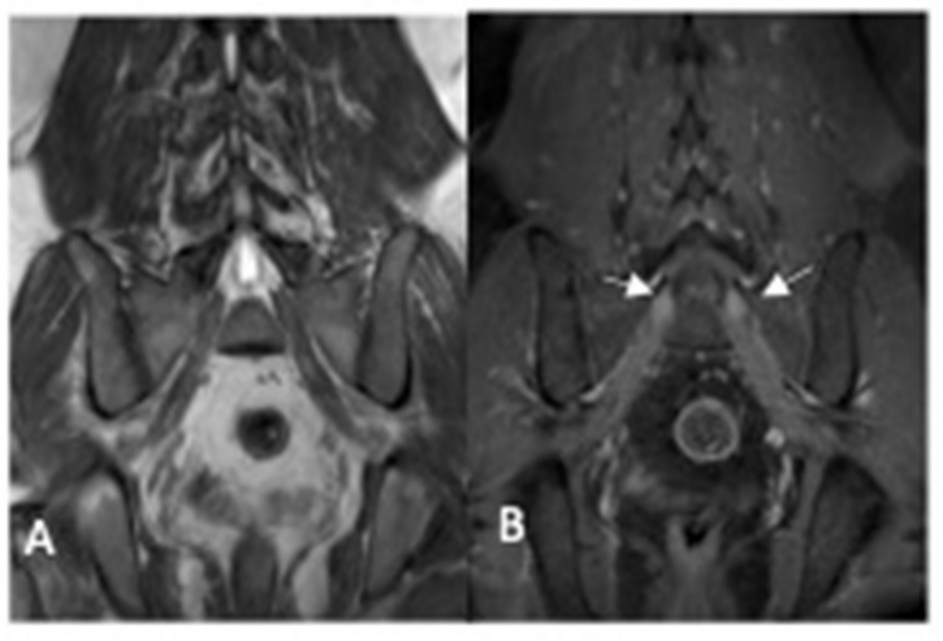
MRI lumbosacral plexus [patient two] showing enlarged sacral nerve roots with post-contrast enhancement (arrow). **(A)** coronol T1 non-contrast image; **(B)** coronal T1 post-contrast image.

CSF analysis was normal. Peripheral nerve ultrasound revealed diffuse and marked nerve enlargement [> x3 ULN] across non-entrapment sites; this was less pronounced across entrapment sites. Colour doppler did not show increase in nerve vascularity. MRI of the lumbar spinal revealed enlargement and enhancement of exiting lumbosacral nerve roots bilaterally (Figure 2). Genetic testing subsequently revealed a duplication of the *PMP22* gene. No other pathogenic variants in genes known to cause Charcot-Marie-Tooth disease were noted. Examination of patient's symptomatic niece and her immediate family was not possible as they reside in a different country.

Patient was initiated on physical therapy but declined the use of foot orthoses. No significant recovery of the bilateral foot drop was observed at the last clinical follow-up, 21 months from onset of neurological symptoms. Final diagnosis was CMT1A with superimposed acute to subacute, atraumatic bilateral common peroneal neuropathies at the fibular neck.

## Discussion

We report two adult CMT1A patients who presented with acute to subacute onset, entrapment neuropathies without known history of compression or trauma, and posit this as a distinct phenotypic variant of CMT1A. The entrapment neuropathies were associated with poor recovery. One patient had mild neurological symptoms since late childhood while another patient was asymptomatic up to the development of multiple entrapment neuropathies. Both patients had a diffuse demyelinating polyneuropathy, with markedly low motor nerve conduction velocities (<20 m/s). In both patients, we demonstrated symptomatic and asymptomatic partial conduction blocks at multiple entrapment sites.

Our cases further expand the spectrum of disease phenotypes associated with CMT1A. While CMT1A is typically characterised as a childhood-onset symmetrical, uniformly demyelinating, gradually progressive polyneuropathy (1), our patients presented with acute/subacute, asymmetric neurological deficits, associated with features of non-uniform demyelination on nerve conduction studies. Mathis et al. and Deymeer et al. had previously reported two patients with acute onset non-specific distal limb symptoms associated with conduction blocks across multiple peripheral nerves (3, 17). These patients may represent variants of acute to subacute atraumatic entrapment neuropathies with CMT1A.

Several differential diagnoses need to be considered when faced with such a clinical presentation. This includes other forms of CMT associated with non-uniform demyelination, Lewis-Sumner syndrome (multifocal acquired demyelinating sensory and motor neuropathy, or MADSAM), as well as overlap hereditary and inflammatory demyelinating neuropathy. Physicians need to be aware of potential pitfalls to avoid misdiagnosis. We discuss ways of differentiating these conditions based on features in history (including family history), physical examination as well as ancillary investigations. A summary of key features associated with these four conditions have been listed in Table 3 (21). Early diagnosis of this condition allows for diagnostic closure, timely genetic counselling and avoidance of costly and potentially toxic immunotherapy.

**Table 3:** Distinguishing features between acute to subacute atraumatic entrapment neuropathies in patients with CMT1A with other CMT associated with non-uniform demyelination, non-uniform demyelinating inherited demyelinating neuropathies, multifocal acquired demyelinating sensory, and motor neuropathy (MADSAM) and coexistent inherited and inflammatory neuropathy.

	**Acute to subacute, atraumatic entrapment neuropathies in patients with CMT1A**	**Other CMT associated with non-uniform demyelination**	**Multifocal acquired demyelinating sensory and motor neuropathy (MADSAM)**	**Overlap inherited and inflammatory demyelinating neuropathy**
**Clinical**				
Age of onset	Younger	Younger	Older (Median age at onset 44 years) (18)	Variable (Mean age at onset 39, range 18–69) (6)
History	Acute/subacute (Initial slow progression may not be noticed by patient)	Gradual (few reports of acute deterioration)	Acute/stepwise	Acute/subacute
Positive sensory symptoms	±	-	+	+
Family history	Yes (family history may not be clear due to phenotypic heterogeneity)	Yes (family history may not be clear if autosomal recessive inheritance pattern/phenotypic heterogeneity)	No	Yes (family history may not be clear due to phenotypic heterogeneity)
Foot deformities	Yes (deformities may be subtle)	Yes (deformities may be subtle)	No	Yes (deformities may be subtle)
Nerve enlargement on palpation	Yes, marked diffuse enlargement	Variable	Yes, focal enlargement	Yes, depending on type on underlying CMT
Weakness	Distal + distribution of entrapment neuropathy (recent change)	Distal	Multifocal and asymmetric distribution	Proximal (recent deterioration)
**CSF**				
CSF protein	Normal or mildly elevated (<1 g/L)	Normal or mildly elevated (<1 g/L)	Elevated CSF protein in 33–82% (18)	Raised with proposed cut-off of >1 g/L (17)
**Nerve conduction study**				
Conduction blocks or temporal dispersion	At entrapment sites more than at non-entrapment sites	At non-entrapment sites more than at entrapment sites	At non-entrapment sites	New conduction blocks on serial studies
Other electrophysiological features	Homogeneous motor nerve conduction velocity slowing between nerves; motor nerve conduction velocity of upper limbs <30 m/s	Variable; motor nerve conduction velocity depends on type on underlying CMT	Heterogeneous motor nerve conduction velocity slowing between nerves	Development of heterogeneous motor nerve conduction velocity slowing between nerves on serial studies
**Nerve magnetic resonance imaging**				
Thickened nerve roots	Yes	Yes	Yes	Yes
Enhancing nerve roots/plexus with gadolinium	Yes	Uncertain	Yes	Yes
**Peripheral nerve ultrasound**	Diffuse marked enlargement with relative sparing of entrapment sites (19)	Variable, normal to slight enlargement (19)	Focal enlargement in areas of demyelination (20)	Uncertain, likely depending on type of underlying CMT
**Response to immunotherapy**	No	No	Yes	Yes

*CMT, Charcot-Marie-Tooth neuropathy; CSF, cerebrospinal fluid*.

Inherited neuropathies tend to present at a younger age as opposed to inflammatory demyelinating neuropathies (22). However, due to the broad phenotypic heterogeneity of CMT1A, there is no strict age cut-off at presentation to guide the diagnosis of acute to subacute atraumatic entrapment neuropathies in patients with CMT1A. All four conditions may present with stepwise or sudden onset, rapidly progressive decline. Acute to subacute development of neurological deficits has been reported in patients with CMT secondary to mutations in the *GJB1, MPZ, SH3TC2, SPTLC1*, and *FIG4* genes (19, 23–29). Careful questioning of prior functional status may reveal a history of a chronic progressive neuropathic process indicative of an underlying inherited polyneuropathy, as seen in patient 1. However, patients with minimal symptoms who have been well-compensated, like patient 2, may not report neuropathic symptoms prior to the acute/subacute entrapment neuropathies.

An autosomal dominant inheritance pattern is a clue toward the diagnosis of an CMT1A. However, a positive family history may not be apparent because of significant phenotypic heterogeneity between affected individuals of different generations, as seen in our patients (4). Lack of family history may occur in a patient with a *de-novo* mutation in the proband as well. Conversely, a positive family history does not exclude an overlap pathology.

The presence of proximal and distal limb involvement represents a polyradiculoneuropathy, commonly associated with an inflammatory demyelinating neuropathy. However, patients can still present with segmental or distal variants of inflammatory demyelinating neuropathy overlapped with an inherited neuropathy potentially making the diagnosis challenging.

Since foot deformities may be subtle, careful physical examination is required to look for features of a chronic neuropathic process. The presence of marked diffuse nerve enlargement as seen in both patients is a prominent feature of CMT1A. On the other hand, Inflammatory demyelinating neuropathy is more likely to result in focal, patchy nerve enlargement (30, 31). Diffuse enlargement of superficial nerves therefore serves as a clue toward the diagnosis of CMT1A, as evidenced by the presence of widespread enlarged superficial nerves in our patients.

While non-uniform demyelination on electrophysiological studies suggests an underlying acquired aetiology, it can be observed in inherited neuropathies as well. Conduction blocks have been rarely reported in CMT1A (3, 17). There are reports of CMT associated with mutations in *GJB1, MPZ, SH3TC2, SPTLC1*, and *FIG4* genes showing partial conduction blocks and temporal dispersion across both entrapment and non-entrapment sites (21–26). It is notable that the reported acute- subacute, atraumatic entrapment neuropathies in patients with CMT1A are predominantly associated with partial conduction blocks across entrapment sites. One must be mindful, however, that criteria-based diagnosis of conduction block may be difficult in nerves with markedly low motor amplitudes. In such situations, needle electromyography may be helpful. Additionally, homogenous motor nerve conduction slowing between different motor nerves examined is seen in acute- subacute, atraumatic entrapment neuropathies in patients with CMT1A. This is in contrast with inflammatory demyelinating neuropathies, where there may be heterogeneous motor nerve conduction velocity slowing between motor nerves.

CSF protein levels may be a helpful adjunct with clinical correlation. While CMT1A may be associated with mild CSF protein elevations, possibly due to nerve root hypertrophy resulting in impaired CSF flow (18), significantly elevated CSF protein of more than 1 g/L is more likely associated with an inflammatory demyelinating neuropathy. Conversely, a normal CSF protein does not definitively exclude an acquired, inflammatory aetiology. Abnormal CSF protein, noted in 33–82% of individuals with MADSAM, illustrates this point (32, 33).

Hauw et al. compared anti-ganglioside antibody positivity of 27 patients with CMT against 32 patients with CIDP. They found 11.1% (3 out of 27) patients with CMT and 3.1% (1 out of 32) patients with CIDP had anti-ganglioside antibodies (22). Anti-ganglioside antibodies are thus likely of limited utility for the differentiation between inherited and inflammatory demyelinating neuropathy. Anti-ganglioside antibodies were not tested in our patients.

Nerve ultrasound serves as an objective modality to quantify degree, extent and sites of nerve enlargement potentially differentiating inherited from acquired causes of hypertrophic neuropathy. Similar to previous reports of CMT1A patients (30), nerve ultrasound findings in our patients demonstrated marked diffuse nerve enlargement, more pronounced at non-entrapment sites compared to entrapment sites. Such findings may be used to distinguish CMT1A from its differentials, since there is slight to no nerve enlargement associated with demyelinating neuropathy secondary to mutations in the *GJB1, MPZ* and *SH3TC2*, while nerve enlargement is found in sites associated with focal demyelination in MADSAM (20). Increased vascularity on doppler had been reported in cases of compressive neuropathy (34). However, we did not note increased vascularity at sites concordant with the acute clinical deficits, possibly due to the delayed nature of the study. Studies evaluating nerve ultrasound studies in the acute phase, following acute neurological worsening, should be pursued. There are no published reports on the utility of nerve ultrasound in patients with overlap hereditary and inflammatory demyelinating neuropathy.

On histopathology, inflammatory demyelinating neuropathies may show the presence of an inflammatory infiltrate as well as focal or multifocal distribution of onion bulb. While an inherited demyelinating neuropathy typically demonstrates the absence of an inflammatory infiltrate as well as a generalised distribution of onion bulbs (21, 35). The role of MR neurography in differentiating between inherited and inflammatory demyelinating neuropathy is uncertain. The presence of gadolinium enhancement of nerve root may also be present in inherited neuropathy without superimposed inflammation (36), as seen in patient two.

The role of using clinical improvement following immunotherapy to differentiate inherited from inflammatory neuropathy is uncertain. Hauw et al. reported 20% (7 out of 35) of patients with CMT experienced clinical improvement following treatment with intravenous immunoglobulin (IVIG) (22). However, objective clinical scores was not used to document clinical improvement and patients with co-existent hereditary and inflammatory neuropathy were not excluded from analysis.

*PMP22* gene expression is under tight regulation and changes in its expression can drastically influence myelination of peripheral nerve fibres (37, 38). Abnormal PMP22 expression is associated with CMT1A and HNPP [hereditary neuropathy with liability to pressure palsy]. It has been observed from animal models that duplication of the *PMP22* gene can cause both dysmyelination of large axons (39), as well as demyelination as a result of Schwann cell instability (40).

Additionally, it has been shown that PMP22 deficiency can lead to the disruption of peripheral nerve myelin tight-junctions. This increases myelin permeability which in turn affects the propagation of axonal action potentials leading to functional demyelination (41). It can be hypothesised that similar pathophysiological processes may occur as the result of the duplication of PMP22 as well.

These processes are likely more pronounced at entrapment sites. This is because entrapment sites have a fixed cross-sectional area which increase the risk of pressure related nerve injury; nerves also experience repeated loading at these sites, leading to greater wear and tear (42). Disproportionate dysmyelination and demyelination in the presence of axonal continuity at entrapment sites can also potential explain why asymptomatic conduction blocks can be observed in patients with acute to subacute atraumatic entrapment neuropathies in patients with CMT1A. The pathophysiology of how mutations in the PMP22 gene lead to disease and phenotypic variability is not fully understood. Phenotypic heterogeneity may be secondary to other genetic as well as epigenetic modifiers, which may hopefully be elucidated through future studies (43).

Our report adds to the phenotypic spectrum of CMT1A. Previous reports of acute-subacute neurological presentations and electrophysiological evidence of non-uniform demyelination have been limited to CMTs associated with the *GJB1, MPZ, SH3TC2, SPTLC1*, and *FIG4* genes (23–29). Our report adds CMT1A to this list, and indicates that more extensive genetic testing, in the form of either panel sequencing or whole exome sequencing, along with other relevant ancillary tests may be beneficial in patients with such a clinical presentation.

There is currently no definitive treatment for acute to subacute atraumatic entrapment neuropathies of CMT1. Patients with clinical and paraclinical evidence suggestive of superimposed inflammatory neuropathy may still benefit from a trial of immunomodulatory treatment. Orthoses can be prescribed to patients with existing neurological deficits. Patients should also be advised on the avoidance of activities that increase the risk of falls as a result of these neurological deficits. As there is potential for the development of significant disability from entrapment neuropathies, additional care by way of avoiding movements and postures that aggravate and provoke entrapment neuropathies should be emphasised to patients. A thorough history and physical examination of at-risk family members would also be helpful so that appropriate medical and genetic counselling can be instituted early in these individuals.

In addition to the small number of patients, there are several other limitations to our study. We did not have normative nerve ultrasound values for CMT1A patients without entrapment neuropathies to serve as a comparator to our patients. Investigations and treatment were performed at the discretion of the treating clinician, a few months from onset of neurological symptoms. Hence investigations were not standardised, and findings during the acute phase of illness could not be assessed. Patient 1 had only received one course of IVIG, and may not be considered as having an appropriate trial of treatment. While the diagnosis of partial conduction block in some nerves with low distal CMAP values was based on published electrophysiological diagnostic criteria, the only additional evidence supporting its diagnosis was derived from qualitative information extrapolated from needle EMG.

In conclusion, we herein describe the clinical features and evaluation of acute to subacute atraumatic entrapment neuropathies in patients with CMT1A and further expand the phenotypic spectrum associated with CMT1A. Multiple clinical and investigative tools, including peripheral nerve ultrasound need to be employed to differentiate this condition from its mimics.

## Data Availability Statement

The original contributions presented in the study are included in the article/Supplementary Material, further inquiries can be directed to the corresponding authors.

## Ethics Statement

The studies involving human participants were reviewed and approved by Singhealth CIRB. The patients/participants provided their written informed consent to participate in this study.

## Author Contributions

ZC, SN, MS, KP, P-SN, JK, and JC: drafting or revising the manuscript for intellectual content. ZC, SN, MS, and JC: study design. ZC, SN, ZP, ML, CK, KT, KP, KV, and JC: acquisition of data. ZC, SN, ZP, KT, KP, KV, SD, WL, and JC: analysis and interpretation of the data. All authors approved the final version of the manuscript.

## Funding

This study was funded by Singapore Health Services (ZC by the SingHealth Precision Medicine Institute (PRISM) grant (AM/PRM009/2020).

## Conflict of Interest

The authors declare that the research was conducted in the absence of any commercial or financial relationships that could be construed as a potential conflict of interest.

## Publisher's Note

All claims expressed in this article are solely those of the authors and do not necessarily represent those of their affiliated organizations, or those of the publisher, the editors and the reviewers. Any product that may be evaluated in this article, or claim that may be made by its manufacturer, is not guaranteed or endorsed by the publisher.

## Abbreviations

CIDP: chronic inflammatory demyelinating polyradiculoneuropathy
CMT: Charcot Marie-Tooth-disease
CSA: cross section area
CSF: cerebrospinal fluid
EMG: electromyography
MADSAM: multifocal acquired demyelinating sensory and motor neuropathy
MRC: Medical Research Council.
