# Supplementary material for: Acute to Subacute Atraumatic Entrapment Neuropathies in Patients With CMT1A: A Report of a Distinct Phenotypic Variant of CMT1A
Source: Front Neurol. 2022 Feb 25;13:826634. doi: 10.3389/fneur.2022.826634 (PMC8914073; doi:10.3389/fneur.2022.826634)
Supplement: Supplementary file 1 [file Data_Sheet_1.pdf]

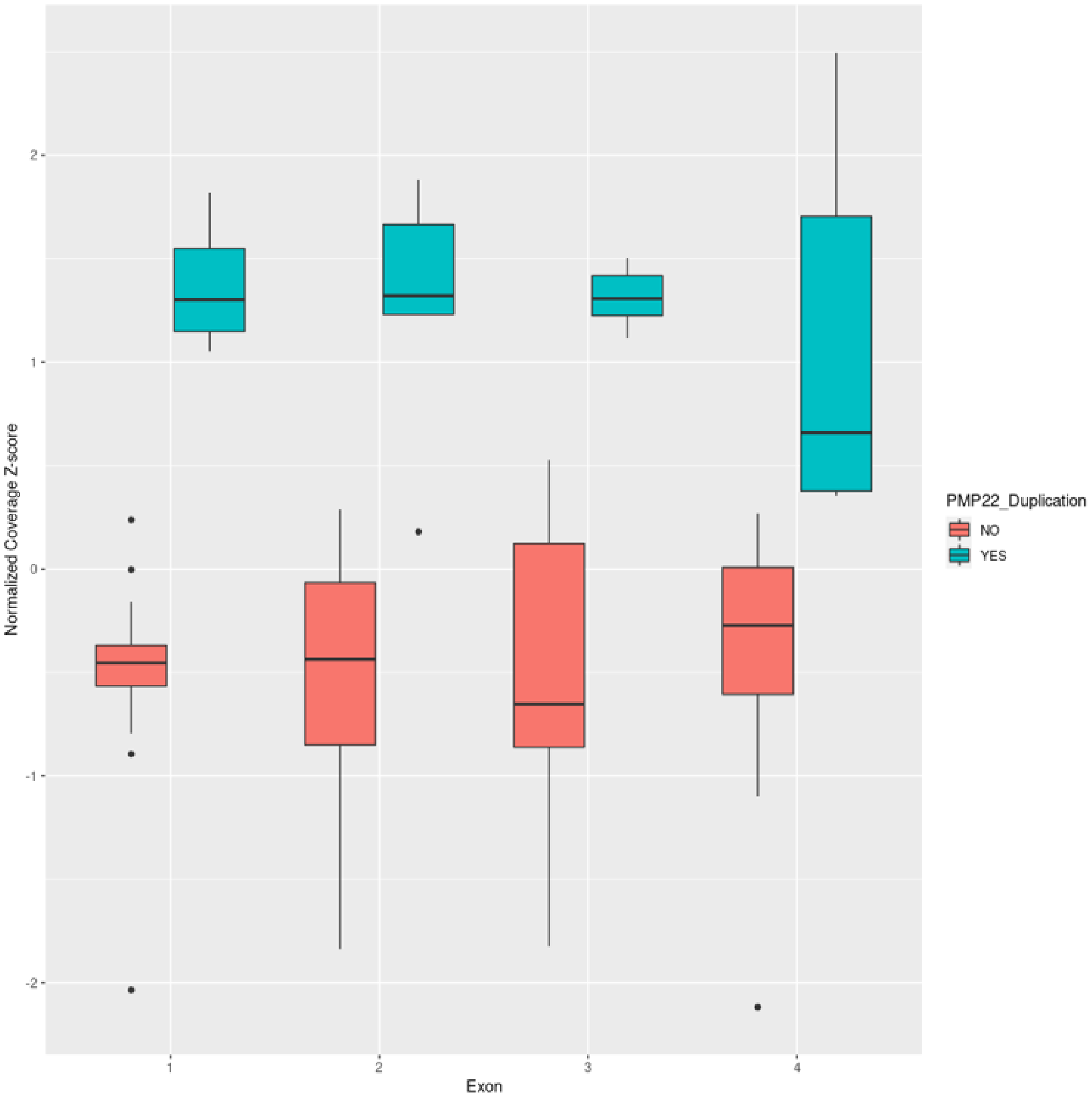

Supplementary figure: Copy Number Variant analysis of PMP22: For each sample, a value showing the coverage median z-score is provided. Green boxes represent samples with median z-score>1, indicating duplications in PMP22. Red boxes represent samples without PMP22 duplications.

			Spontaneous Activity		Motor Unit Potential					
**Muscle**	**Side**	**Insertion Activity**	**Fibs**	**PSW**	**Polyphasia**	**Amplitude**	**Duration**	**Activation**	**Recruit-ment pattern**	**Remarks**
Tibialis anterior	Left	Increased	2+	2+	Normal	Reduced	Reduced	Normal	Discrete	Severe acute denervation changes with discrete motor unit recruitment
Biceps short head	Left	Increased	1+	1+	Normal/1+	Normal	Normal	Normal	Slightly reduced	Mild acute on chronic denervation changes with slight reduction in motor unit recruitment
Gastrocnemius medial head	Left	Increased	1+	1+	Normal/1+	Normal/1+	Prolonged	Normal	Slightly reduced	Mild acute on chronic denervation with slight reduction in motor unit recruitment
Vastus lateralis	Left	Increased	1+	1+	Normal/1+	Normal/1+	Normal	Normal	Slightly reduced	Mild acute on chronic denervation changes
L5 paraspinal	Left	Normal	None	None						Normal
First dorsal interossei	Left	Increased	1+	1+	Normal	Increased	Prolonged	Normal	Discrete	Severe acute-on-chronic denervation changes
Flexor carpi ulnaris	Left	Increased	None	None	Normal	Normal/1+	Prolonged	Normal	Reduced	Severe acute-on-chronic denervation changes
Extensor digitorum communis	Left	Increased	None	None	1+	Normal	Normal	Normal	Slightly reduced	Mild acute on chronic denervation changes
Biceps brachii	Left	Normal	None	None	Normal	Normal	Normal	Normal	Complete	Normal
Triceps	Left	Normal	None	None	Normal	Normal/1+	Normal	Normal	Complete	Normal
Brachioradialis	Left	Increased	None	None	1+	Normal/1+	Normal	Normal	Slightly reduced	Mild acute on chronic denervation changes

Supplementary Table 1. Needle EMG findings for patient 2: Needle EMG findings for patient 2: Needle EMG changes are in keeping with disproportionate denervation localised to the left common peroneal nerve proximal to the left tibialis anterior and distal to the short head of the left biceps femoris (likely at the left fibular neck). This corresponds to the patient's neurological finding of a left foot drop. Needle EMG of the left medial gastrocnemius, a muscle innervated by a nerve of similar length compared to that of the left tibialis anterior only shows mild denervation changes with slight reduction in motor unit recruitment.

[Fibs, fibrillation potentials; PSW, positive sharp waves]

	Minimal Temporal Dispersion (duration increases by 30% or less)				Moderate Temporal Dispersion (duration increased by 31% to 60%)	
Nerve Segment (proximal/distal)	Definite Partial Conduction Block		Probable Partial Conduction Block		Probable Partial Conduction Block	
	Amplitude Reduction	Area Reduction	Amplitude Reduction	Area Reduction	Amplitude Reduction	Area Reduction
**Median**						
Forearm (E/W)	>50%	>40%	40% to 49%	30% to 39%	>50%	>40%
Arm (AX/E)	>50%	>40%	40% to 49%	30% to 39%	>50%	>40%
**Ulnar**						
Forearm (BE/W)	>50%	>40%	40% to 49%	30% to 39%	>50%	>40%
Across Elbow (AE/BE)	>50%	>40%	40% to 49%	30% to 39%	>50%	>40%
Arm (AX/AE)	>50%	>40%	40% to 49%	30% to 39%	>50%	>40%
**Radial**						
Forearm (E/DF)	-	-	>50%	>40%	>60%	>50%
Arm (AX/E)	-	-	>50%	>40%	>60%	>50%
Proximal (EP/AX)	-	-	>50%	>40%	>60%	>50%
**Peroneal**						
Leg (BF/ankle)	>60%	>50%	50% to 59%	40% to 49%	>60%	>50%
Across FH (AF/BF)	>50%	>40%	40% to 49%	30% to 39%	>50%	>40%
**Tibial**						
Leg (knee/ankle)	>60%	>50%	50% to 59%	40% to 49%	>60%	>50%

Supplemental table 2: Criteria for the diagnosis of definite and probable conduction block. Based on the 1999 Guidelines in Electrodiagnostic Medicine. American Association of Electrodiagnostic Medicine[9]

AE = Above Elbow; AF = Above Fibular Head; BE = Below Elbow; BF = Below Fibular Head; DF = Distal Forearm; E = Elbow; FA = Forearm; FH = Fibular Head W = Wrist

AARS	EGR2	IGHMBP2	MORC2	RAB7A
AIFM1	FBLN5	INF2	MPZ	SBF1
ARHGEF10	FGD4	KIF5A	MTMR2	SBF2
ATP1A1	FIG4	LITAF	NDRG1	SGPL1
BAG3	GARS	LMNA	NEFH	SH3TC2
BSCL2	GDAP1	LRSAM1	NEFL	SLC25A46
COX6A1	GJB1	MARS	PDK3	SPG11
DHTKD1	GNB4	MCM3AP	PLEKHG5	SURF1
DNAJB2	HARS	MED25	PMP2	TFG
DNM2	HINT1	MFN2	PMP22	TRIM2
DRP2	HSPB1	MICAL1	PRPS1	TRPV4
DYNC1H1	HSPB8	MME	PRX	YARS

Supplemental Table 3. List of genes known to cause Charcot-Marie-Tooth disease evaluated in patients 1 and 2
